# Robotic-Assisted Kinematically Aligned Total Knee Arthroplasty Demonstrated Early Rehabilitation and Select Mental Health-Related Quality of Life Improvements Compared to Conventional MA-TKA

**DOI:** 10.3390/jcm15124817

**Published:** 2026-06-21

**Authors:** Jiawei Chen, Katelyn Kaye-Ling Lim, Hong Yu Jared Chua, Jeremy Tze En Lim, Nicolaas C. Budhiparama, Seng Jin Yeo, Ming Han Lincoln Liow

**Affiliations:** 1 Department of Orthopaedic Surgery, Singapore General Hospital, Singapore Health Services, Singapore 169608, Singapore; 2 Department of Orthopaedic and Traumatology, Faculty of Medicine, Universitas Airlangga, Surabaya 60115, Indonesia; 3 Department of Orthopaedics, Leiden University Medical Centre, 2333 ZA Leiden, The Netherlands; 4 Nicolaas Institute of Constructive Orthopaedic Research & Education Foundation for Arthroplasty & Sports Medicine at Medistra Hospital, Jakarta 12950, Indonesia

**Keywords:** mechanical, kinematic, VELYS^TM^, ATTUNE^TM^, functional/early outcomes

## Abstract

**Introduction:** Currently, there is an ongoing debate regarding the benefits of kinematic alignment (KA) versus mechanical alignment (MA) in total knee arthroplasty (TKA). Robotic-assisted TKA has been shown to improve implant positioning and precision of the KA technique, enabling successful kinematic alignment. However, its impact on early postoperative and functional outcomes remains unclear. This study aims to examine how imageless, table-mounted, robotic-assisted KA-TKA compares with conventional MA-TKA. **Methods:** Registry data of all primary TKAs using ATTUNE™ cruciate-retaining implants (January 2021–December 2024) performed by a single, experienced surgeon in a high-volume arthroplasty center were retrospectively reviewed. A total of 64 patients who underwent robotic-assisted KA-TKA were compared to 39 patients who underwent conventional MA-TKA. The mean age was 70.3 ± 7.71 and 69.3 ± 9.47 in the KA-TKA group and the MA-TKA group, respectively, while the male proportion was 32.8% and 30.7%, respectively. Early postoperative outcomes (static/dynamic pain score, ambulation distance, length of stay) and 6-month functional outcomes (range of motion, Knee Society Score, Oxford Knee Score, SF-36, patient expectation/satisfaction scores) were analyzed. Delta changes in outcome scores and proportion of patients attaining a minimum clinically important difference (MCID) were studied. **Results:** Robotic-assisted KA-TKA displayed benefits in the majority of the early postoperative outcomes, with significant improvements in ambulation distance (23.3 vs. 14.7 m, *p* = 0.002) compared to conventional MA-TKA. Both groups showed significant improvements in the majority of the functional outcomes at 6 months. Robotic-assisted KA-TKA also shows significant improvements in selected mental health aspects of SF-36, namely vitality (*p* = 0.001), mental health (*p* = 0.048), mental component summary (MCS) (*p* = 0.004), and a larger proportion attaining SF-36 vitality MCID (*p* = 0.045). Following false discovery rate correction for multiple comparisons, postoperative ambulation distance, SF-36 vitality, and MCS remained statistically significant between groups. No significant differences in KSS, OKS, and satisfaction/expectation fulfillment were noted. **Conclusions:** Robotic-assisted KA-TKA demonstrated early rehabilitation and select mental health-related quality of life improvements compared to conventional MA-TKA. Further studies are needed to examine its long-term clinical outcomes.

## 1. Introduction

Total Knee Arthroplasty (TKA) is the gold-standard treatment for patients suffering from advanced knee osteoarthritis (OA), of which multiple alignment philosophies exist [1]. The mechanical alignment (MA) technique restores the patient’s knee alignment to neutral by positioning implants perpendicular to the distal femoral and proximal tibial mechanical axis. Although this technique has demonstrated long-term implant survivorship and reproducibility, it may not preserve the patient’s natural anatomy and has been associated with patient dissatisfaction despite radiographic success [2,3,4].

On the other hand, kinematic alignment (KA) is a technique that aims to restore a patient’s pre-arthritic joint lines and native limb alignment by adopting a ligament-sparing technique. Based on the patient’s pre-arthritic kinematic axes, it relies mainly on bony cuts to achieve patient-specific limb alignment and knee biomechanics [5]. By preserving the native femoral and tibial joint obliquity and rotation, KA-TKA restores more natural knee kinematics and potentially delivers better functional outcomes. Multiple studies have highlighted the advantages of KA compared to MA [6], including greater patient satisfaction, quicker rehabilitation, improved range of motion, and enhanced gait mechanics [5,7]. Nonetheless, debate remains regarding implant-positioning accuracy and durability with KA [8,9]. A network meta-analysis comparing various assistive technologies showed that robotic-assisted TKA was most likely to achieve implant position accuracy, followed by computer navigation, patient-specific instrumentation, and lastly conventional techniques [10]. While the study focuses on mechanical alignment due to limited KA-specific data, its applicability to the KA-TKA remains uncertain.

There has been an increase in the interest in robotic-assisted TKAs (RA-TKAs) as a means of improving implant positioning accuracy and reproducibility [11,12]. The RA-TKA also shows enhanced precision in bone resections, soft tissue releases, and balancing, and facilitates intraoperative execution of personalized alignment strategies, which may be particularly relevant in KA-TKA where restoration of patient-specific anatomy is intended. A variety of such RA-TKA systems exists, including image-based and imageless systems, with their outcomes studied in comparison to their conventional counterparts. VELYS^TM^ robotic-assisted solution (VRAS), a recent advancement in the field of image-less RA-TKA, facilitates intraoperative planning by providing precise cartilage mapping for the femur and anatomical reference points for tibial resection, enabling accurate execution for surgeons using KA with the ATTUNE™ knee system [12,13]. This technology could potentially address the limitations of the conventional KA approach; however, it remains uncertain how robotic-assisted KA-TKA outcomes measure up to those of conventional MA-TKA, which is still commonly performed and supported by extensive data.

This study aims to assess early postoperative recovery and 6-month functional outcomes in patients undergoing VELYS^TM^ (DePuy Synthes, Johnson & Johnson MedTech, Warsaw, NJ, USA) robotic-assisted KA-TKA compared to conventional MA-TKA. Areas of focus include postoperative ambulation, pain, length of stay, and 6-month patient-reported outcome measures (PROMS) in terms of physical and psychological wellness, to determine whether robotic-assisted KA-TKA is beneficial from the patient’s perspective. We hypothesize that the patients who underwent VELYS^TM^ robotic-assisted KA-TKA would demonstrate improved early post-operative and short-term patient-reported outcome measures compared to those in the conventional MA-TKA group.

## 2. Materials and Methods

### 2.1. Study Design

We conducted a retrospective cohort study on patients who underwent total knee arthroplasty in our institution. Registry data of patients who underwent primary total knee arthroplasty using ATTUNE^TM^ implant from January 2021 to December 2024, either with VELYS^TM^ robotic-assisted KA-TKA or conventional MA-TKA, were retrospectively reviewed. An initial 107 patients met the inclusion criteria, 4 of whom were excluded from the study due to missing early postoperative outcome data. A total of 103 patients were identified for analysis, including 64 patients who underwent robotic-assisted KA-TKA and 39 patients who underwent conventional MA-TKA. Inclusion and exclusion criteria can be found in Table 1. Patient’s demographic parameters, such as age, race, height, BMI, and male proportion, were recorded. Early-term and 6-month postoperative outcomes were retrospectively analyzed.

During the study period, the VRAS was introduced into practice at the start of 2024, representing a transition from conventional instrumentation to robotic-assisted surgery. Therefore, from 2024 onwards, all patients receiving the ATTUNE^TM^ implant underwent robotic-assisted KA-TKA, while prior cases were performed using conventional MA-TKA. As such, treatment allocation was determined by time period and not randomisation.

The Centralized Institutional Review Board and Ethics Committee’s approval was sought before the commencement of the project (CIRB: 2024-4046). Patient data were stored in the institution’s knee registry, which was retrospectively extracted and anonymised for analysis.

### 2.2. Surgical Technique

All surgeries were performed by a single, fellowship-trained surgeon in a high-volume arthroplasty center performing over 2500 knee replacement surgeries annually, for both robotic-assisted and conventional groups. All patients received ATTUNE^TM^ Knee system (DePuy Synthes, Johnson & Johnson MedTech, Warsaw, NJ, USA) cruciate-retaining implants. Conventional TKA followed an MA protocol for component alignment. A standardized technique using a combination of measured resection and gap balancing in extension and flexion using spacer blocks was performed according to the manufacturer’s instrumentation instructions to achieve balanced flexion and extension gaps.

VELYS^TM^ Robotic-Assisted Solution (software version 1.8, DePuy Synthes, Johnson & Johnson MedTech, Warsaw, NJ, USA) was used in all robotic-assisted procedures and completed without robot malfunction or the need for conversion to the manual technique. A standard midline skin incision and medial parapatellar arthrotomy were performed initially. The patella was then everted, and selective excision of periarticular soft tissues was performed to better visualize the knee joint. The lateral aspect of the anterior fat pad was exposed to facilitate anterior femoral registration. Femoral and tibial tracking pins were inserted intra-incisionally, and the navigation arrays were firmly secured to their respective bony structures. Bone resections were tailored according to a restrictive KA technique, with 7 mm and 9 mm resections for the distal medial and lateral femur, respectively, accounting for implant thickness and an assumed 2 mm cartilage loss. In cases of pronounced varus deformity, where the preoperative medial proximal tibial angle was markedly reduced (i.e., <83°), a maximum varus correction threshold of 7° was applied to the tibial cut. The native posterior tibial slope was targeted but capped at 7° for all cases.

Patients in both groups underwent patelloplasty, lateral facetectomy, and removal of patella osteophytes, without patellar resurfacing. Dilute betadine irrigation was performed before the capsule was closed. This was followed by administration of local periarticular analgesia. Tranexamic acid was applied topically before the capsule and skin were closed. A pneumatic tourniquet was used throughout the surgery. No wound drainage was used after capsule and skin closure. All patients received postoperative physiotherapy and standard wound care according to institutional guidelines.

### 2.3. Clinical Evaluation

At our institution, physiotherapists examined patients pre-operatively and 6 months postoperatively by evaluating the range of motion (flexion and extension), Oxford Knee Score (OKS), Knee Society knee and function scores (KSS), and Short-Form 36 (SF-36) health survey questionnaire. The knee range of motion was assessed by senior physiotherapists using a goniometer. Outcome evaluators were not blinded to treatment allocation, and patients were aware of the specific treatment they received.

The OKS includes 12 questions that evaluate one’s pain and function. Each question is scored from 1 to 5, where 1 indicates the best and 5 the worst outcome, with total scores ranging from 12 (best) to 60 (worst) [14].

The KSS knee and function scores assess objective (pain, knee alignment, ROM) and subjective (function, i.e., walking, stairs, use of walking aid) outcomes [15]. Each score ranges from 0 to 100, with higher scores reflecting better outcomes.

The SF-36 health survey assesses eight domains: physical function (PF), role physical (RP), bodily pain (BP), general health (GH), vitality (VI), social function (SF), role emotional (RE), and mental health (MH). Each domain is rated from 0 to 100, with higher scores equating to better outcomes. These domains can be further categorized into the physical component summary (PCS) and mental component summary (MCS) [16].

The proportion of patients achieving the minimum clinically important difference (MCID) was calculated for both robotic-assisted KA-TKA and conventional MA-TKA groups. Six-month MCID thresholds were obtained from current literature and included the following: OKS (5 points), KSS function score (10 points), KSS knee score (9 points), SF-36-PF (11.6 points), SF-36-RP (11.7 points), SF-36-BP (16.9 points), SF-36-GH (0.85 points), SF-36-VI (3.9 points), SF-36-SF (11.7 points), SF-36-RE (7.7 points), SF-36-MH (4 points), SF-36 PCS (10 points) and MCS (10 points) [17,18,19,20].

Patient satisfaction and fulfillment of expectations were assessed at 6 months postoperatively. Satisfaction is scored from 1 to 7, while fulfillment of expectations is scored from 1 to 6. Scores of 1 to 3 on both scales indicate patient satisfaction and expectations fulfilled, while scores above 3 indicate dissatisfaction and unfulfilled expectations.

On postoperative day 1 (POD1), inpatient physiotherapists guided patients through standard mobilization exercises such as knee bend, static quads, inner-range quads, and straight-leg raise. During this session, both static and dynamic pain scores were assessed using the Visual Analog Scale (VAS), as well as the ambulation distance. Along with surgical duration and length of stay, these measures comprise the early postoperative outcomes evaluated in this study.

### 2.4. Statistical Analysis

Statistical analysis was conducted using RStudio (Version 2022.12.0+353, Posit, PBC, Boston, MA, USA). A post hoc power analysis was conducted using a power of 0.8 and a significance of 0.05. SF-36 Vitality and MCS were used as representatives of PROMs as they reflect broader functional and psychosocial recovery following TKA. The required sample size was 28 and 39 for SF-36 vitality and MCS, respectively. Given a sample size of 64 in the robotic-assisted KA-TKA group and 39 in the conventional MA-TKA, this study’s sample size was likely sufficient for these outcomes. However, given the limitations of the post hoc power analysis, findings should be interpreted in the context of observed effect sizes and their corresponding 95% confidence intervals (CI), which is more precise and relevant clinically.

Continuous data were presented as mean and standard deviation. Statistical significance was defined as *p* ≤ 0.05, which is the convention for type I errors. Prior to analysis, assumptions for parametric testing were assessed. Normality of continuous variables was evaluated using the Shapiro–Wilk test and visual inspection of histograms, while homogeneity of variances between groups was assessed using Levene’s test. As these assumptions were reasonably satisfied, the student’s unpaired *t*-test was used to compare quantitative variables between both groups. The mean difference between groups and 95% CI were recorded. For within-group comparisons over time (e.g., pre-operative and 6 months), paired *t*-tests were used. A chi-squared test for proportions was used to compare categorical variables (e.g., satisfaction/expectation, proportion of patients meeting MCID). To account for multiple comparisons across various outcomes, the false discovery rate (FDR) using the Benjamini–Hochberg method was used for multiplicity control.

## 3. Results

### 3.1. Demographics

**Figure 1:**
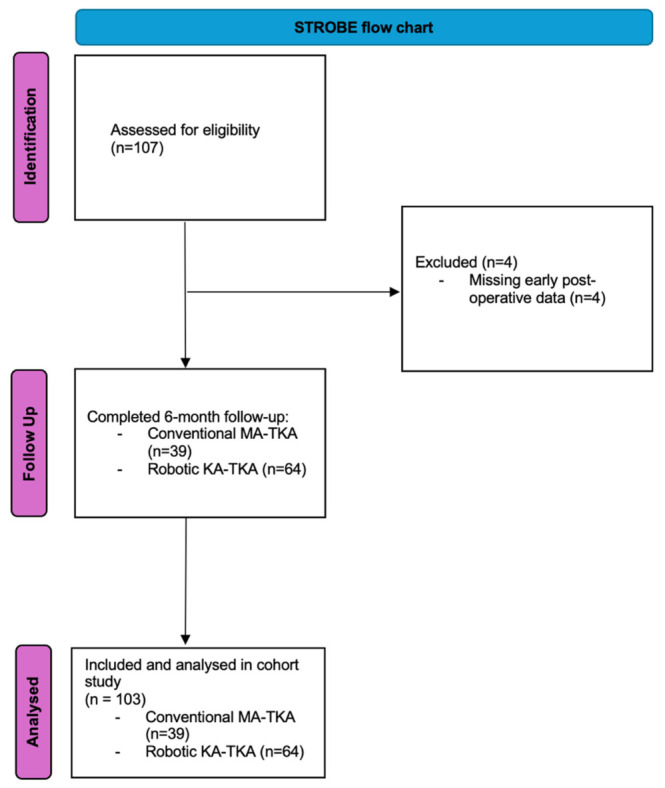
STROBE flow chart.

An initial 107 patients who met the inclusion criteria were extracted from the institution’s registry data. Four patients were excluded from the study due to missing early postoperative outcome data. A total of 64 patients in the robotic-assisted KA-TKA group and 39 patients in the conventional MA-TKA group had completed 6-month follow-up and were available for analysis, as shown in Figure 1 according to the STROBE flow chart. No significant differences were observed between groups in terms of gender proportions (*p* = 1), mean BMI (*p* = 0.419), age of patients (*p* = 0.564), or any common patient comorbidities which may confound outcomes (e.g., diabetes, hypertension, hyperlipidemia, concomitant arthropathies or spine conditions) (Table 2).

Both robotic-assisted KA-TKA and conventional MA-TKA groups showed significant improvements in the majority of the functional outcome scores at 6 months compared to pre-operatively (from *p* < 0.001 to 0.008). However, the conventional MA-TKA group had a larger proportion of functional outcomes not displaying any significant change at 6 months (SF-36 general health, vitality, role emotional, mental health, and MCS) (Table 3).

### 3.2. Early Postoperative Outcomes

The robotic KA-TKA group showed a significant improvement in POD1 ambulation distance compared to conventional MA-TKA (23.3 ± 18.5 m vs. 14.7 ± 8.51 m, *p* = 0.002). No significant differences were observed between both groups in terms of surgical duration, static and dynamic pain scores, and time to discharge (*p* = 0.401, 0.472, 0.489, and 0.494, respectively) (Table 4).

### 3.3. Six-Month Functional Outcomes

At 6 months, range of motion (flexion and extension), OKS, KSS function, and knee scores showed no significant differences between groups (Table 5). The robotic-assisted KA-TKA group showed significant differences in SF-36 vitality (81.6 ± 14.6 vs. 68.1 ± 21.9, mean difference 13.5, 95% CI 5.66–21.5, *p* = 0.001), mental health (87.9 ± 14.6 vs. 81.6 ± 15.6, mean difference 6.3, 95% CI 0.07–12.4, *p* = 0.048) and mental component summary (58.9 ± 8.5 vs. 53.0 ± 10.2, mean difference 5.9, 95% CI 1.95–9.75, *p* = 0.004), while the rest of the SF-36 outcome scores showed no significant differences between groups (Table 6).

Additionally, the robotic-assisted KA-TKA group had a significantly bigger proportion of patients achieving SF-36 vitality MCID (56.3 vs. 35.9%, mean difference 20.4%, 95% CI 1.0–39.7%, *p* = 0.045) (Table 7). Differences in the other domains of proportion achieving MCID, as well as 6-month satisfaction and expectation fulfillment, were not statistically significant (Table 8).

### 3.4. Multiplicity Control

**Figure 2:**
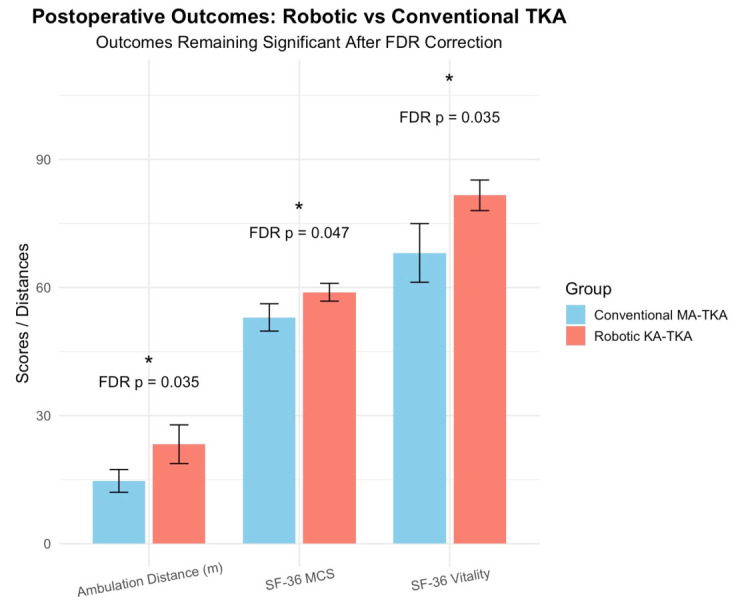
Bar graph of statistically significant postoperative outcomes after FDR adjustment. * statistical significance (*p* < 0.05).

Due to the presence of multiple outcomes of comparison, FDR using the Benjamini–Hochberg method was applied to adjust for any type-I error inflation. After adjustment for multiple comparisons, statistically significant differences were observed in ambulation distance (*p* = 0.035), SF-36 Vitality (*p* = 0.035), and SF-36 MCS scores (*p* = 0.047) between the two groups. Although SF-36 MH and SF-36 Vitality MCID demonstrated statistical significance on unadjusted analysis (*p* = 0.048 and 0.045, respectively), these findings did not remain statistically significant after FDR adjustment (*p* = 0.336 and 0.336, respectively), suggesting that these findings may reflect chance rather than true differences and should therefore be interpreted cautiously. The full FDR adjustment results can be found in Supplementary Materials. The postoperative outcomes that remained statistically significant after FDR adjustment can be found in Figure 2, together with their respective *p*-values.

## 4. Discussion

The main findings of this study include improved POD1 ambulation distance and select 6-month mental health-related quality of life improvements in the robotic-assisted KA-TKA group, as seen through significant differences in SF-36 vitality, mental health, MCS, and proportion of patients achieving SF-36 vitality MCID. Their corresponding between-group difference and 95% CI generally favored the robotic-assisted KA-TKA group, although only selected outcomes (ambulation distance, SF-36 vitality, and MCS) remained statistically significant after FDR adjustment. Several confidence intervals (e.g., SF-36 MH and vitality MCID) were especially close to the null value, suggesting a modest effect size and potentially contributing to its loss of significance following multiplicity control. Additionally, robotic-assisted KA-TKA showed no statistically significant difference to conventional MA-TKA in other early outcomes such as POD1 pain, surgical duration, length of stay, and functional outcomes such as OKS, KSS function, and knee scores, and physical aspects of SF-36. These results were observed on the background of similar patient baseline demographics such as age, gender, BMI, and patient comorbidities.

Enhancements in mobility postoperatively are an important aspect in the patient recovery journey, reducing associated complications from prolonged immobility [21,22]. By respecting native joint kinematics and reducing the frequency of soft-tissue releases to preserve the patient’s native soft-tissue envelope, KA may aid in reducing pain, improving range of motion, and therefore promoting earlier and enhanced ambulation [23]. When supplemented by the precision of robotics as provided by the VELYS^TM^ system, the benefits of KA may be further amplified through accurate execution [24]. Multiple studies comparing other robotic systems with conventional TKA show improvements in early post-operative outcomes, including reduced post-operative pain and a decreased length of stay [25,26,27]. In our present study, ambulation distance was the only early postoperative outcome that reached statistical significance. Other outcomes, such as pain score, length of stay, and surgical duration, showed no significant differences between groups. A total of 56.3% of robotic-assisted RA-TKA patients and 48.7% of conventional MA-TKA patients reported no static pain (VAS score of 0) postoperatively, while the proportion was 50% and 56.4% for mild to no dynamic pain (VAS score of 0–2), respectively. The large number of patients reporting the lowest possible pain scores postoperatively may potentially contribute to a floor effect, limiting any significant differences. While the comparable length of stay and surgical duration may be influenced by standardized institutional workflows, including discharge criteria and operating theater workflow, comparability in surgical duration may also be attributed to the surgeon’s experience on the robotic platform after overcoming its initial learning curve [28].

The KA-TKA represents a distinct alignment philosophy compared to MA-TKA, with multiple meta-analyses showing non-inferiority of the two techniques [29,30,31]. A meta-analysis of level 1 studies comparing KA-TKA using patient-specific instrumentation (PSI) to MA-TKA showed no significant differences between groups regarding functional and pain outcomes [32]. Conversely, our present study utilizing VELYS^TM^ robotic-assistance for KA-TKA highlights possible selected functional benefits. Doan et al. (2022) and Alton et al. (2025) have shown VELYS’s ability to achieve desired accuracy and reduce outliers over conventional instrumentation across various alignment strategies of the robotic-assisted group [13,33]. Given the selected improvements in mental health-related quality of life outcomes in our present study, these findings may indicate a potential role of VELYS^TM^ robotic assistance in supporting a surgeon in achieving individualized KA. Despite non-significant improvements in traditional functional scores like OKS and KSS, it is important to note that these scoring systems focus primarily on mechanical function and pain during daily activities. On the other hand, SF-36 consists of broader aspects of a patient’s health-related quality of life, including mental well-being and energy levels. Significant improvements in patients’ SF-36 vitality, mental health, and MCS in the robotic-assisted KA-TKA group may reflect broader patient-perceived benefits in health-related quality of life. Its sensitivity in capturing benefits outside of the traditional physical outcome scores may suggest robotic-assisted KA-TKA’s association with aspects of postoperative recovery not entirely captured in traditional outcome measures [34]. However, it is also important to note that certain SF-36 outcomes that were nominally significant before multiplicity control, such as the SF-36 MH and SF-36 Vitality MCID, did not remain significant after FDR correction. Hence, these findings may be affected by type I error inflation and should be interpreted as exploratory. Nevertheless, the observed improvements in SF-36 Vitality and MCS, along with post-operative ambulation distance, may potentially indicate meaningful functional and psychosocial benefits associated with robotic-assisted KA-TKA.

Despite statistical significance in between-group differences, the clinical relevance of these findings should be interpreted cautiously. Certain observed differences were modest in their extent and may not necessarily result in clinically meaningful improvements observed at the patient level. Whilst robotic-assisted KA-TKA showed favorable differences in selected SF-36 domains, there were no statistically significant differences in the MCID of outcomes after FDR adjustment, which reflects changes in a clinical intervention that are meaningful for a patient. Clinically, this may also be reflected by a patient’s satisfaction and fulfillment of expectations after the operation, which also did not significantly differ between groups. Nevertheless, the observed improvements in postoperative ambulation distance, SF-36 Vitality, and SF-36 MCS may still reflect potentially meaningful differences in selected aspects of early recovery and patient-perceived health-related quality of life.

Strengths of this study include (1) a comparative study of cases by a single surgeon, reducing confounding brought about by minor variations between different surgeons, (2) the use of the same implant across both groups, and (3) the use of multiple validated knee functional scores. This study is not without its limitations. Firstly, due to the retrospective nature of this study and the nature of the assessors and patients being unblinded to treatment type, selection bias and assessor bias may be introduced. This may especially influence subjective PROMs, including psychological and mental health-related outcomes within the SF-36 domains, where expectation and placebo-related effects may contribute to perceived differences between groups. However, analysis of baseline demographics such as gender proportions, BMI, age, and patient comorbidities showed no significant differences, thereby reducing the risk of confounding. Secondly, radiographic outcomes and complications were not assessed in this study. Therefore, although prior studies have shown robotic-assisted systems to be associated with improving implant positioning and reducing outliers, the relationship between implant alignment accuracy and clinical outcomes could not be directly evaluated in our present study. Additionally, improvements in postoperative outcomes may not be entirely attributable to the use of robotic-assistance or alignment philosophy alone, as there may be other patient factors that may influence these outcomes, such as education level, socioeconomic status, and level of social support. Nonetheless, in the context of similar patient comorbidities, this may be an early indication of improvements in selected early postoperative outcomes associated with the robot’s accuracy and consistency in reducing outliers in KA. Thirdly, with the current generation of robotics, fewer surgeons are aiming for mechanical alignment. The presence of two variables in our study—alignment philosophy and use of robotic assistance—precludes a direct comparison of the effects of robotics or KA alone. Furthermore, the lack of randomization and the temporal separation between both groups may introduce time-related bias associated with evolving perioperative management, rehabilitation pathways, surgical workflow, and increasing team experience. Fourthly, pre-operative ambulation distance and pain scores were not available for analysis, which would provide insight into delta improvements in patient function. Although pre-operative PROMs such as KSS function score and SF-36 PF were comparable between groups, we cannot exclude potential confounding by unmeasured functional status. Therefore, the observed differences in POD1 ambulation distance should be interpreted as associations rather than definitive proof of superior early functional recovery. Lastly, given the recency of VRAS and its adoption in the institution, there is a limited number of patients with 6-month functional outcomes, and long-term outcomes at 2 years and beyond are not available. Robotic-assisted KA-TKA’s impact on long-term outcomes and survivorship is left to be studied. However, none of the patients in the present cohort required repeated operations or revisions at 6 months.

## 5. Conclusions

In our retrospective cohort study, VELYS^TM^ robotic-assisted KA-TKA was associated with early post-operative ambulation and select mental health-related quality of life improvements compared to conventional MA-TKA. Despite similarities in traditional patient-reported outcomes such as OKS and KSS, select psychological and mental health-related improvements in the robotic-assisted KA-TKA group, particularly postoperative ambulation distance, SF-36 MH, and MCS following multiplicity correction, may suggest potential patient-perceived benefits. Further studies are needed to examine its long-term clinical outcomes.

## Figures and Tables

**Table 1:** Inclusion and exclusion criteria.

Inclusion Criteria	Exclusion Criteria
Patients undergoing primary TKA	Patients undergoing revision TKA
Patients undergoing TKA for primary knee OA	Patients undergoing TKA for indications other than primary knee OA
Use of either the VELYS robotic system with the ATTUNE implant or the conventional ATTUNE implant	Use of robotic systems other than VELYS, or use of non-ATTUNE implants
Documentation of early outcome data (e.g., surgical duration, post-operative pain scores, length of stay, and ambulation distance) and pre-operative and 6-month functional outcomes (e.g., KSS, SF-36)	Missing early outcome data, pre-operative or 6-month functional data (e.g., from lost-to-follow-up)

**Table 2:** Baseline patient demographics. BMI and age are reported as mean ± standard deviation.

	Kinematic Robotic	Mechanical Conventional	Significance/*p*-Value
Total no. of knees (patients)	64	39	
*Gender*			
Male	21	12	1 (n.s.)
Female	43	27	
BMI/kg m^−2^	27.8 ± 4.40	28.5 ± 3.90	0.419 (n.s.)
Age/years	70.3 ± 7.71	69.3 ± 9.47	0.564 (n.s.)
*Co-morbids*			
Diabetes Mellitus	20	7	0.208 (n.s.)
Hypertension	41	27	0.747 (n.s.)
Hyperlipidemia	43	29	0.584 (n.s.)
Concomitant Arthropathies (e.g., gout, rheumatoid arthritis)	6	2	0.688 (n.s.)
Spine conditions (e.g., cervical myelopathy, lumbar stenosis	5	2	0.903 (n.s.)

n.s.—non-significant.

**Table 3:** Functional outcomes at pre-op and 6 months for the robotic KA-TKA (*n* = 64) and conventional MA-TKA (*n* = 39) groups.

Functional Outcomes	Kinematic Robotic			Conventional Mechanical		
	Pre-op	6 Months	*p*-Value	Pre-op	6 Months	*p*-Value
Range of motion						
Extension	4.52 ± 5.22	2.27 ± 4.48	0.002	5.31 ± 5.33	1.97 ± 3.81	0.003
Flexion	115.9 ± 19.6	115.0 ± 15.7	0.556 (n.s.)	118.1 ± 16.3	113.2 ± 12.5	0.0558 (n.s.)
Oxford Knee Score (OKS) *	36.5 ± 8.94	20.7 ± 7.29	(<0.001)	36.6 ± 7.32	22.1 ± 7.41	(<0.001)
KSS function score	50.1 ± 22.5	68.6 ± 26.2	(<0.001)	45.8 ± 23.0	62.4 ± 28.5	(<0.001)
KSS knee score	37.0 ± 16.0	84.6 ± 13.3	(<0.001)	40.2 ± 16.7	81.4 ± 14.2	(<0.001)
SF-36 physical function	37.0 ± 26.4	65.2 ± 28.8	(<0.001)	36.8 ± 21.0	59.9 ± 30.8	(<0.001)
SF-36 role physical	12.9 ± 29.9	64.8 ± 42.7	(<0.001)	9.62 ± 24.7	55.1 ± 45.2	(<0.001)
SF-36 bodily pain	30.0 ± 16.3	68.0 ± 25.6	(<0.001)	31.6 ± 17.1	63.1 ± 30.3	(<0.001)
SF-36 general health	69.3 ± 16.7	75.7 ± 15.4	0.008	63.1 ± 21.6	69.8 ± 18.3	0.104 (n.s.)
SF-36 vitality	72.5 ± 19.4	81.6 ± 14.6	(<0.001)	69.4 ± 21.2	68.1 ± 21.9	0.707 (n.s.)
SF-36 social function	52.3 ± 35.7	86.1 ± 29.5	(<0.001)	50.0 ± 32.8	78.5 ± 33.9	(<0.001)
SF-36 role emotional	93.2 ± 24.6	97.9 ± 13.1	0.182 (n.s.)	99.1 ± 5.34	97.4 ± 11.8	0.421 (n.s.)
SF-36 mental health	82.7 ± 15.9	87.9 ± 14.6	0.004	84.0 ± 14.3	81.6 ± 15.6	0.413 (n.s.)
SF-36 PCS	30.9 ± 8.11	45.4 ± 10.4	(<0.001)	31.6 ± 7.29	44.5 ± 10.9	(<0.001)
SF-36 MCS	53.2 ± 10.1	58.9 ± 8.52	(<0.001)	51.5 ± 11.0	53.0 ± 10.2	0.413 (n.s.)

* OKS—lower scores indicate better outcomes. n.s.—non-significant.

**Table 4:** Comparison of early post-operative outcomes between kinematic and mechanical TKA groups (surgical duration, static and dynamic pain scores, ambulation distance, and time to discharge).

	Kinematic Robotic		Mechanical Conventional		Mean Difference	95% CI of Mean Difference	*p*-Value
	Mean ± SD	MoE	Mean ± SD	MoE			
Surgical duration/min	69.3 ± 12.5	3.12	71.9 ± 16.8	5.44	2.6	−8.84 to 3.59	0.401 (n.s.)
Pain score (VAS)							
Static	1.11 ± 1.63	0.408	1.36 ± 1.74	0.564	0.25	−0.937 to 0.438	0.472 (n.s.)
Dynamic	3.56 ± 2.03	0.507	3.26 ± 2.24	0.728	0.3	−0.571 to 1.18	0.489 (n.s.)
Ambulation distance/m	23.3 ± 18.5	4.63	14.7 ± 8.51	2.76	8.6	3.26 to 13.9	**0.002**
Time to Discharge/days	2.03 ± 1.72	0.429	2.26 ± 1.65	0.534	0.23	−0.910 to 0.443	0.494 (n.s.)

MoE—Margin of Error (half-width of 95% CI). n.s.—non-significant. Bold values indicate statistical significance (*p* < 0.05).

**Table 5:** Comparison of pre-operative and 6-month functional outcomes between kinematic and mechanical TKA groups (ROM, OKS, KSS).

	Kinematic Robotic		Mechanical Conventional		Mean Difference	95% CI of Mean Difference	*p*-Value
	Mean ± SD	MoE	Mean ± SD	MoE			
*Range of Motion*							
*Extension*							
Pre-op	4.52 ± 5.22	1.30	5.31 ± 5.33	1.73	0.79	−2.93 to 1.35	0.463 (n.s.)
6 months	2.27 ± 4.48	1.12	1.97 ± 3.81	1.27	0.3	−1.38 to 1.96	0.728 (n.s.)
*Flexion*							
Pre-op	115.9 ± 19.6	4.88	118.1 ± 16.3	5.29	2.2	−9.35 to 4.87	0.532 (n.s.)
6 months	115.0 ± 15.7	3.92	113.2 ± 12.5	4.18	1.8	−3.79 to 7.53	0.513 (n.s.)
*Oxford Knee Score (OKS)* *							
Pre-op	36.5 ± 8.94	2.23	36.6 ± 7.32	2.37	0.1	−3.40 to 3.03	0.908 (n.s.)
6 months	20.7 ± 7.29	1.82	22.1 ± 7.41	2.40	1.4	−4.34 to 1.61	0.365 (n.s.)
*KSS function score*							
Pre-op	50.1 ± 22.5	5.61	45.8 ± 23.0	7.44	4.3	−4.90 to 13.5	0.355 (n.s.)
6 months	68.6 ± 26.2	6.54	62.4 ± 28.5	9.23	6.2	−5.02 to 17.3	0.276 (n.s.)
*KSS knee score*							
Pre-op	37.0 ± 16.0	4.00	40.2 ± 16.7	5.41	3.2	−9.88 to 3.42	0.337 (n.s.)
6 months	84.6 ± 13.3	3.31	81.4 ± 14.2	4.72	3.2	−2.45 to 8.94	0.260 (n.s.)

* OKS—lower scores indicate better outcomes. MoE—Margin of Error (half-width of 95% CI). n.s.—non-significant.

**Table 6:** Comparison of pre-operative and 6 months functional outcomes between kinematic and mechanical TKA groups (SF-36).

	Kinematic Robotic		Mechanical Conventional		Mean Difference	95% CI of Mean Difference	*p*-Value
	Mean ± SD	MoE	Mean ± SD	MoE			
*SF-36 physical function*							
Pre-op	37.0 ± 26.4	6.59	36.8 ± 21.0	6.82	0.2	−9.12 to 9.59	0.960 (n.s.)
6 months	65.2 ± 28.8	7.19	59.9 ± 30.8	9.98	5.3	−6.79 to 17.5	0.382 (n.s.)
*SF-36 role physical*							
Pre-op	12.9 ± 29.9	7.46	9.62 ± 24.7	8.02	3.3	−7.54 to 14.1	0.549 (n.s.)
6 months	64.8 ± 42.7	10.7	55.1 ± 45.2	14.7	9.7	−8.19 to 27.6	0.283 (n.s.)
*SF-36 bodily pain*							
Pre-op	30.0 ± 16.3	4.08	31.6 ± 17.1	5.54	1.6	−8.35 to 5.25	0.651 (n.s.)
6 months	68.0 ± 25.6	6.40	63.1 ± 30.3	9.81	4.9	−6.7 to 16.5	0.404 (n.s.)
*SF-36 general health*							
Pre-op	69.3 ± 16.7	4.18	63.1 ± 21.6	7.00	6.2	−1.83 to 14.3	0.128 (n.s.)
6 months	75.7 ± 15.4	3.85	69.8 ± 18.3	5.95	5.9	−1.11 to 12.9	0.098 (n.s.)
*SF-36 vitality*							
Pre-op	72.5 ± 19.4	4.83	69.4 ± 21.2	6.86	3.1	−5.15 to 11.4	0.453 (n.s.)
6 months	81.6 ± 14.6	3.64	68.1 ± 21.9	7.09	13.5	5.66 to 21.5	**0.001**
*SF-36 social function*							
Pre-op	52.3 ± 35.7	8.92	50.0 ± 32.8	10.6	2.3	−11.4 to 16.1	0.735 (n.s.)
6 months	86.1 ± 29.5	7.36	78.5 ± 33.9	11.0	7.6	−5.48 to 20.7	0.250 (n.s.)
*SF-36 role emotional*							
Pre-op	93.2 ± 24.6	6.15	99.1 ± 5.34	1.73	5.9	−12.3 to 0.449	0.068 (n.s.)
6 months	97.9 ± 13.1	3.28	97.4 ± 11.8	3.83	0.5	−4.49 to 5.45	0.848 (n.s.)
*SF-36 mental health*							
Pre-op	82.7 ± 15.9	3.97	84.0 ± 14.3	4.63	1.3	−7.33 to 4.70	0.666 (n.s.)
6 months	87.9 ± 14.6	3.65	81.6 ± 15.6	5.05	6.3	0.072 to 12.4	**0.048**
*SF-36 PCS*							
Pre-op	30.9 ± 8.11	2.03	31.6 ± 7.29	2.36	0.7	−3.77 to 2.37	0.652 (n.s.)
6 months	45.4 ± 10.4	2.59	44.5 ± 10.9	3.53	0.9	−3.43 to 5.21	0.684 (n.s.)
*SF-36 MCS*							
Pre-op	53.2 ± 10.1	2.53	51.5 ± 11.0	3.57	1.7	−2.59 to 6.07	0.426 (n.s.)
6 months	58.9 ± 8.52	2.13	53.0 ± 10.2	3.32	5.9	1.95 to 9.75	**0.004**

n.s.—non-significant. Bold values indicate statistical significance (*p* < 0.05).

**Table 7:** Comparison of the proportion achieving MCID at 6 months between kinematic and mechanical groups.

Functional Score	Kinematic Robotic (%)	Conventional Mechanical (%)	95% CI (%)	*p*-Value
Oxford Knee Score (5) *	89.1	89.7	0.186 to 3.98 (OR)	1 (n.s.)
*Knee Society Score*				
Function Score (10)	73.4	66.7	−0.116 to 0.251	0.463 (n.s.)
Knee Score (9) *	98.4	89.7	0.666 to 359 (OR)	0.067 (n.s.)
*Short-Form 36*				
Physical function (11.6)	65.6	51.3	−0.052 to 0.339	0.149 (n.s.)
Role physical (11.7)	68.8	59.0	−0.094 to 0.289	0.313 (n.s.)
Bodily pain (16.9)	76.6	66.7	−0.082 to 0.280	0.274 (n.s.)
General health (0.85)	60.9	59.0	−0.176 to 0.215	0.844 (n.s.)
Vitality (3.9)	56.3	35.9	0.01 to 0.397	**0.045**
Social function (11.7)	57.8	59.0	−0.208 to 0.185	0.908 (n.s.)
Role emotional (7.7) *	6.3	2.6	0.237 to 128 (OR)	0.647 (n.s.)
Mental health (4)	54.7	38.5	−0.032 to 0.337	0.11 (n.s.)
PCS (10)	62.5	61.5	−0.184 to 0.203	0.922 (n.s.)
MCS (10)	29.7	23.1	−0.107 to 0.239	0.465 (n.s.)

* Proportion of MCID for OKS, KSS—knee score, and SF-36 role emotional scores is analyzed with the Fisher exact test, and their 95% CI is presented as odds ratio (OR) due to small cell count < 5. n.s.—non-significant. Bold values indicate statistical significance (*p* < 0.05).

**Table 8:** Satisfaction and fulfillment of expectations at 6 months.

	Kinematic (n = 64)	Mechanical (n = 39)	Significance/*p*-Value
Satisfied (%)	95.1	94.9	1 (n.s)
Dissatisfied (%)	4.9	5.1	
Expectations fulfilled (%)	93.4	87.2	0.306 (n.s)
Expectations not fulfilled (%)	6.6	12.8	

n.s.—non-significant.

## Data Availability

The data that support the findings of this study are available on reasonable request from the corresponding author. The data are not publicly available due to privacy or ethical restrictions.

## Supplementary Materials

The following supporting information can be downloaded at https://www.mdpi.com/article/10.3390/jcm15124817/s1: Table S1: False detection rate.

## Abbreviations

The following abbreviations are used in this manuscript:

TKA	Total Knee Arthroplasty
rTKA	Robotic Total Knee Arthroplasty
cTKA	Conventional Total Knee Arthroplasty
KA	Kinematic Alignment
MA	Mechanical Alignment
OA	Osteoarthritis
VRAS	VELYS^TM^ Robotic-assisted System
OKS	Oxford Knee Score
KSS	Knee Society Score
SF-36	Short-Form 36
PCS	Physical Component Summary
MCS	Mental Component Summary
BMI	Body Mass Index
MCID	Minimal Clinically Important Difference
POD1	Post-operative day 1
VAS	Visual Analog Scale
FDR	False Discovery Rate
