# Supplementary material for: Robotic-Assisted Kinematically Aligned Total Knee Arthroplasty Demonstrated Early Rehabilitation and Select Mental Health-Related Quality of Life Improvements Compared to Conventional MA-TKA
Source: J Clin Med. 2026 Jun 21;15(12):4817. doi: 10.3390/jcm15124817 (PMC13301004; doi:10.3390/jcm15124817)
Supplement: Supplementary file 1 [file jcm-15-04817-s001.zip › jcm-4318557-supplementary.pdf]

Table S1. False detection rate.

Outcome	Raw_p	Adjusted_p
SF36 Vitality (6m)	0.001	0.035
Ambulation distance	0.002	0.035
SF36 MCS (6m)	0.004	0.046667
SF36 Vitality MCID	0.045	0.336
SF36 MH (6m)	0.048	0.336
KSS knee MCID	0.067	0.390833
SF36 GH (6m)	0.098	0.48125
SF36 MH MCID	0.11	0.48125
SF36 PF MCID	0.149	0.579444
SF36 SF (6m)	0.25	0.684688
KSS knee (6m)	0.26	0.684688
SF36 BP MCID	0.274	0.684688
KSS function (6m)	0.276	0.684688
SF36 RP (6m)	0.283	0.684688
Expectation	0.306	0.684688
SF36 RP MCID	0.313	0.684688
OKS (6m)	0.365	0.690577
SF36 PF (6m)	0.382	0.690577
Surgical duration	0.401	0.690577
SF36 BP (6m)	0.404	0.690577
KSS function MCID	0.463	0.690577
SF36 MCS MCID	0.465	0.690577
Static pain	0.472	0.690577
Dynamic pain	0.489	0.690577
Time to discharge	0.494	0.690577
Flexion ROM	0.513	0.690577
SF36 RE MCID	0.647	0.838704
SF36 PCS (6m)	0.684	0.855
Extension ROM	0.728	0.878621
SF36 GH MCID	0.844	0.957419
SF36 RE (6m)	0.848	0.957419
SF36 SF MCID	0.908	0.977879
SF36 PCS MCID	0.922	0.977879
OKS MCID	1	1
Satisfaction	1	1
